# The influence of one-time biofeedback electromyography session on the firing order in the pelvic floor muscle contraction in pregnant woman–A randomized controlled trial

**DOI:** 10.3389/fnhum.2022.944792

**Published:** 2022-09-29

**Authors:** Monika Błudnicka, Magdalena Piernicka, Jakub Kortas, Damian Bojar, Barbara Duda-Biernacka, Anna Szumilewicz

**Affiliations:** ^1^Department of Clinical Physiotherapy and Professional Practices, Gdansk University of Physical Education and Sport, Gdańsk, Poland; ^2^Department of Sport, Gdansk University of Physical Education and Sport, Gdańsk, Poland; ^3^Department of Health and Life Sciences, Gdansk University of Physical Education and Sport, Gdańsk, Poland

**Keywords:** pelvic floor muscle onset, firing order, biofeedback EMG, pelvic floor, pregnancy, pelvic floor muscle training, exercise, women

## Abstract

Many women are initially unable to contract the pelvic floor muscles (PFMs) properly, activating other muscle groups before, or instead of, PFM. Numerous authors have proved that biofeedback can be an ideal tool supporting learning of the PFM contraction. However, there is currently a lack of scientific data on how many biofeedback sessions are necessary in this educational process. In this study we aimed at assessing the effects of one-time electromyography (EMG) biofeedback session on the order in which PFM are activated (so called firing order) during conscious contractions in relation to selected synergistic muscles in pregnant, continent women. A randomized controlled trial was conducted in 90 healthy nulliparous women with uncomplicated pregnancies and without diagnosed urinary incontinence. We divided the participants into a biofeedback group (50) and a control group (40). They were, respectively: 30 ± 4 and 30 ± 4 years old, at their 23 ± 5 or 25 ± 7 week of gestation and presented 23 ± 5 or 24 ± 5 kg/m^2^ BMI value (M + SD). Surface EMG with vaginal probes has been used to assess the PFM firing order in selected tasks: in five 3-s maximal contractions (quick flicks), five 10-s contractions, and in a 60-s contraction (static hold). We used the 1–5 scale, where “1” meant the best score, awarded when PFM was activated first in order. The most important finding of our study is that a single EMG biofeedback substantially improved the PFM contractions in pregnant women. First, when applying one-time biofeedback session, more women maintained correct technique or improved it in the second assessment, compared to the control group (73 vs. 65%). Secondly, using the quantitative and qualitative analysis with the Chi-square McNamara B/C test, in the biofeedback group we observed a statistically significant improvement of PFM firing order in four tasks: in the first quick flicks (*p* = 0.016), third quick flicks (*p* = 0.027), fifth quick flicks (*p* = 0.008), and in the first 10-s contractions (*p* = 0.046). In the control group we observed better outcome only in one motor task: in the fourth 10-s contraction (*p* = 0.009). Given the positive effects of a single session of EMG biofeedback on the firing order in the PFM contractions, it should be recommended for pregnant women without urinary incontinence to teach them how to perform PFM exercises correctly.

## Introduction

Pelvic floor muscle (PFM) disorders occur when the muscles or connective tissues of the pelvic area weaken or become damaged. Pregnancy is one of the stressors of the PFMs, which may lead to common PFM disorders, including urinary incontinence (UI), fecal incontinence (FI), pelvic organ prolapse (POP), and sexual dysfunction (DeLancey, 2016; Aoki et al., 2017; Eickmeyer, 2017; Dumoulin et al., 2018; Dornowski et al., 2018; Chmielewska et al., 2019; Cornelia et al., 2019; Nunes et al., 2019; Soave et al., 2019). Proper PFM training plays a vital role in preventing pelvic floor dysfunctions (Devreese et al., 2004; Batista et al., 2011; Mørkved and Bø, 2014; DeLancey, 2016; Aoki et al., 2017; Dumoulin et al., 2018; Moser et al., 2018; Chmielewska et al., 2019; Nunes et al., 2019; Soave et al., 2019). Therefore, it is recommended for pregnant women by various sports medicine and health organizations, including World Health Organization (WHO, 2020).

In PFM training, as in any training for other muscle groups, the correct technique is a key aspect. During the activation of pelvic-floor muscles, there should be a minimum possible activity of the synergistic muscles (especially abdominals) to minimize intraabdominal pressure. According to Mørkved and Bø (2014) simultaneous contractions of outer and more commonly used larger muscle groups outside the pelvis may mask the awareness and strength of the pelvic-floor muscle contraction. Even a strong pelvic-floor muscle contraction following the activation of the abdominals may not be enough to stop urine leakage (Szumilewicz et al., 2019b). The timing of pelvic-floor muscle activity in relation to the activity of other trunk muscles seems to be a crucial factor in maintaining continence (Moser et al., 2018; Koenig et al., 2021).

In recent years, the methods of teaching PFM contractions in women have become the subject of research by scientists and discussions by practitioners. Ben Ami and Dar (2018) compared the effectiveness of four different verbal instructions in correct contractions of PFM, examining the displacement of the pelvic floor by transabdominal ultrasound. They observed that the most effective verbal instruction for correct activation of the PFM was the instruction of “squeezing the anus.” The majority (90%) of participants succeeded in correct contraction of the PFM (Ben Ami and Dar, 2018). Kandadai et al. (2015) and Vermandel et al. (2015) stated that women do not need special equipment to fully understand the concept of correct PFM contractions. On the other hand, some authors underlined that initially women might be unable to contract PFM properly (Kandadai et al., 2015; Vermandel et al., 2015; Szumilewicz et al., 2019b). Therefore, some of them may need external support to achieve better outcomes, especially in the therapies of stress UI or other pelvic floor dysfunctions (Wu et al., 2021).

Biofeedback (BF) has been used for more than 50 years in rehabilitation to facilitate normal movement patterns of PFM after injury and after childbirth (Giggins et al., 2013). It can be referred to as augmented or extrinsic feedback, which provides the user with additional information, above and beyond the information that is naturally available as opposed to the sensory (or intrinsic) feedback that provides self-generated information to the user from various intrinsic sensory receptors (Giggins et al., 2013). What’s more, unlike any other physiotherapeutic technique, biofeedback delivers to patients biological information in real-time. In our previous study, we described the properties of biofeedback techniques used in PFM training, developed for healthy pregnant women (Szumilewicz et al., 2019b). The most commonly used technique of biofeedback were: palpation, the use of perineometer, ultrasonography, electrostimulation, and electromyography (EMG).

The advantages of surface EMG with vaginal probes are that the PFM assessment is non-invasive, painless, the patients is fully dressed during the assessment and the equipment gives information both on the level of neuromuscular activity of particular muscle groups and on the time-based parameters. One of these parameters is the so called “firing order,” which indicates the order in which the PFM are activated: whether the PFM are activated first or whether the synergistic muscles support or disrupt the work of PFM. Therefore, vaginal EMG biofeedback is a common tool that researchers and clinicians use to examine any potential changes in PFM activity (Vermandel et al., 2015) and to teach women how to correctly perform PFM exercises (Szumilewicz et al., 2019b). The EMG PFM assessments with vaginal probes showed acceptable test–retest reliability and clinical predictive validity for use in the prevention and early detection of PFM disorders in previous studies (Glazer et al., 1999). Recently, Scharschmidt et al. (2020) proved the similarity of the intrasession, intraday, and from day to day reliability of results obtained using a probe with circumferential electrode-position to the data collected with longitudinally oriented bars.

Based on the literature review (Bludnicka et al., 2019) we concluded that the biofeedback interventions aimed at improving PFM function varied substantially in terms of intervention time, and also in terms of the frequency and number of biofeedback sessions. Until now, other authors focused mainly on multiple repetition of biofeedback (Batista et al., 2011; de Oliveira Ferro et al., 2020; Kopanska et al., 2020). We raised a question, whether one-time EMG biofeedback session could be beneficial for pregnant, continent women. In our previous study performed in the same research group we observed that a single EMG biofeedback session improved the level of neuromuscular activity in conscious PFM contractions (Bludnicka et al., 2020). The significant improvement in the EMG amplitude was recorded in 10- and 60-s contractions. In this study, we aimed at assessing the effect of one-time biofeedback in pregnant, continent women on the firing order of PFM in relation to selected synergistic muscles in various PFM contractions.

## Materials and methods

The target group for this study were healthy nulliparas (*n* = 90) with uncomplicated pregnancies (age 30 ± 4 years, 21 ± 5 weeks of gestation; M ± SD). Eligibility criteria included: no contraindications to physical activity, ability to undergo the PFM assessment with a vaginal probe and absence of allergic reactions to the materials used during the study. Our primary goal was to ensure patient safety and to provide accurate data for the analysis; therefore, the exclusion criteria were any current or previous pelvic floor dysfunction diagnosed by healthcare professionals. In addition, women who did not have a good quality of life according to the Incontinence Impact Questionnaire short version (IIQ) were also excluded from the study. UI can alter the ability to activate the PFMs (Koenig et al., 2021), so it was important to screen the study women in this regard. The IIQ is useful to quantify quickly the UI-related life-impact. Previous psychometric studies on the IIQ, using classical test theory methods, demonstrated good internal consistency and test–retest reliability of this tool (Monticone et al., 2021). According to the study by Corcos (Corcos et al., 2002), a score less than or equal to 50 on the IIQ scale meets the criteria for a “good quality of life.” A score between 50 and 70 indicates “moderate quality of life” and any score above 70 is classified as “poor quality of life.” Only women with “good quality of life” with IIQ scores below 50 scores were included in the analysis.

Recruitment was continuous and women were randomly assigned to a biofeedback or control groups (depending on the order in which they volunteered for the study). In the statistical analyses, we included data from 50 participants from the biofeedback group and from 40 women from the control group. Some women resigned from the study after receiving information that they had been assigned to the control group (hence their smaller number compared to the biofeedback group). Due to the specifics of the study (women either received visual biofeedback or they did not), it was not possible to blind the participants to the group allocation. Nevertheless, the laboratory worker assessing the contractions of PFM and statisticians were blinded to the group allocation.

**FIGURE 1:**
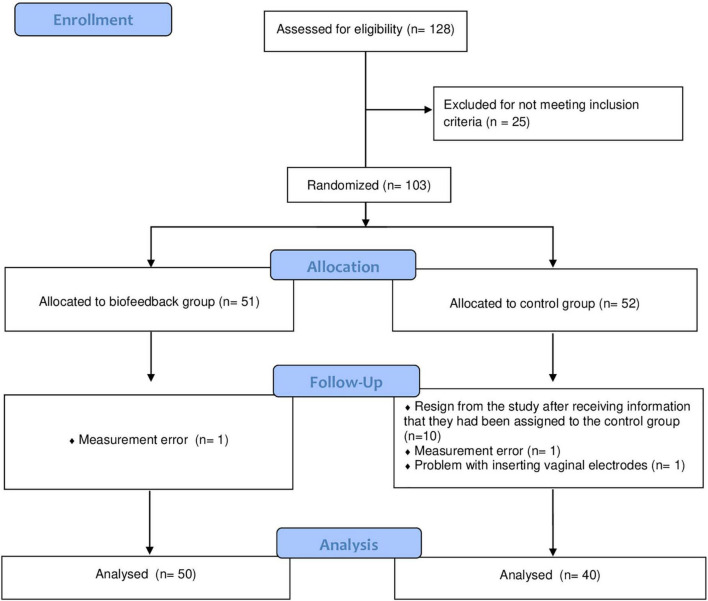
The flow of participants through the study.

The participant flow through the study is shown in Figure 1.

This research was conducted as a part of the ISRCTN. DOI: 10.1186/ISRCTN92265528: “PFM training with surface EMG” project. The study was retrospectively registered on 25 July 2016 with regard to the pilot phase that we carried out in 2013–2015. For the later stages of the project, including the data presented in this paper (completed on 31 May 2019), this was a prospective registration.

The trial was carried out in the Laboratory of Physical Effort and Genetics in Sport, at Gdansk University of Physical Education and Sport in Poland. The principles of the WMA Declaration of Helsinki were used as guidelines for this study and the approval of the Bioethics Commission at the District Medical Chamber in Gdansk, Poland (KB–8/14) was obtained beforehand. The participants were asked to sign informed consent prior to the commencement of PFM assessments.

### Assessment of neuromuscular activity of pelvic floor muscles and implementation of a biofeedback session

During a visit at the laboratory, the participant performed two consecutive PFM assessments using surface EMG. The study was carried out according to the SENIAM standards in terms of EMG signals recording and processing. The EMG signal was registered with 16-bit accuracy at a sampling rate of 1,500 Hz using the TeleMyo™ 2400T direct transmission system (DTS), NORAXON EMG and Sensors System (Scottsdale, AZ, USA). For the further proceeding of the EMG signals we used the MyoResearch XP Master Edition 1.08.32 software that was designed by the manufacturer to support the above equipment. The EMG data was filtered using the built-in hardware high pass filter set to 10 Hz. The raw EMG data were visually checked for artifacts.

The purpose of the study was to analyze neuromuscular activity and the firing order of the PFMs and synergistic muscles. The EMG is painless and non-invasive for the study participants (Glazer et al., 1999). For the PFM assessment we used vaginal probes (Lifecare PR-02, Everyway Medical Instruments Co., Ltd., Taiwan), which are easy to applicate and comfortable for the study participants: weight: 23.1 g; length: 76 mm; diameter: 28 mm. The probes consist of two longitudinal recording plates on both sides, made of stainless steel and nickel. The probes were placed intravaginally by the participants themselves with each plate pointed toward their hips. Halski et al. (2013) observed that different probe placements during the PFM contractions have not impacted the sEMG evaluation in any way, allowing the tested women to place it according to their preferences and comfort. Surface disk electrodes (SKINTACT Premier W-60, LEONHARD LANG GmbH, Austria) were applied for following synergistic muscles: rectus abdominis, obliquus, externus, abdominis, and gluteus maximus. All EMG assessments were carried out in the same laboratory under the supervision of a professional physiotherapist experienced in the field of the urogynecology and in the use of surface EMG.

The second assessment of the biofeedback group involved watching their muscles being activated on a computer screen. Four circles were displayed on a screen, each one representing a different muscle group in the body: the PFM, the rectus abdominis muscles, the oblique abdominals and the gluteal muscles. On the “Relax” command, the circles should enlarge and on the “Contract” command, they should shrink or disappear. The control group did not have the opportunity to observe changes on the monitor during the second EMG assessment.

In this study, we evaluated the firing order of PFM in relation to synergistic muscles using the following 1–5 scale: (1) PFM activated first in order (the most beneficial technique of the PFM contraction); (2) PFM activated second; (3) PFM activated third; (4) PFM activated fourth in order; (5) lack of PFM neuromuscular activity. To compare the differences in the changes in pelvic floor neuromuscular activity between the groups and between the two consecutive assessments we analyzed the scores obtained in the following motor tasks: in five 3-s maximal contractions (quick flicks), in five 10-s contractions and the scores obtained in the 60-s static hold.

The MyoResearch XP software allowed the technique assessor to determine two things: first, whether the particular muscles were activated or not (based on the EMG threshold for muscle onset), and second, the order in which they were activated (measured on a metric timescale to muscle onset), i.e., the “firing order.” Hodges and Bui (1996) claim that reliable assessment of EMG onset is possible with the use of computer-based algorithmic calculations. In our study, the moment of muscle onset was determined automatically with the MyoResearch software option, based on the calculations of the standard deviation range of the EMG baseline before a certain activity and using an appropriate multiplication factor. We measured the PFMs’ EMG baseline for 10 s. During each motor task, a muscle was considered to be activated when its activity during a contraction exceeded the triple standard deviation range of the EMG baseline. To avoid interference in the data analysis by single EMG spikes, we chose a sub-period time of 0.2 s where the EMG signal had to continuously remain above the threshold. All EMG curves were assessed by the technique assessor to support the choice of the SD multiplication factor (Hodges and Bui, 1996) and detect potential data disturbances by artifacts.

### Classification of the study participant as responders and non-responders

Based on the performance of PFM contractions during the first and second EMG assessments we classified our participants as “Responders” and “Non-responders.” “Responders” were these study women who presented correct technique at the first assessment (they activated the PFM first) and maintained the same technique until the second assessment. As “responders,” we also qualified those women who initially failed to activate the PFM first in order but did it properly in the second assessment. The “non-responders” term applied to participants who presented incorrect technique in both EMG assessments and/or presented worse technique in the second assessment (they activated synergistic muscles before PFMs or were not able to activate PFMs at all).

### Statistical analysis

Statistical analysis was performed using Statistica 13.1 software. All values are expressed as mean (M) ± standard deviation (SD). The baseline differences in selected variables between groups were analyzed with the Mann–Whitney *U* test. The analysis of variance (ANOVA Friedman’s test) and Dunn–Bonferroni *post hoc* tests were performed to identify significantly different results in the firing order during PFM contractions between the two study groups after the intervention (as intervention we treated the second performance of EMG assessment with or without biofeedback). To better visualize the distribution of the technique of PFM contractions between consecutive EMG assessments within the individual groups, the results were assigned to the contingency table. The Chi-square McNamara B/C test was used to compare the numbers of participants preforming correct or incorrect technique of PFM contractions in the first and second assessments in the biofeedback and control groups, separately. The statistical significance was set at *p* < 0.05.

## Results

In the Table 1 we have presented the characteristics of the study groups. The biofeedback group and the control group did not statistically differ in terms of age, week of pregnancy, BMI and symptoms of pelvic floor disorders assessed with the IIQ (IIQ score). The first assessment revealed no statistically significant differences in the PFM firing order in analyzed motor tasks between groups.

**TABLE 1:** Characteristics of the study groups.

Variable at baseline	All participants; *n* = 90	Biofeedback group; *n* = 50	Control group; *n* = 40	*p*-value*
Age, years	30 ± 4	30 ± 4	30 ± 4	0.16
Gestational age, weeks	24 ± 6	23 ± 5	25 ± 7	0.22
BMI, kg/m^2^	24 ± 4	23 ± 5	24 ± 5	0.50
IIQ score	1 ± 2	0 ± 1	1 ± 3	0.42
**The firing order of the pelvic floor muscles in the following motor tasks in the:**				
first quick flicks	2.58 ± 1.95	2.44 ± 1.94	2.75 ± 1.97	0.43
second quick flicks	2.69 ± 1.99	2.60 ± 1.98	2.80 ± 2.02	0.69
third quick flicks	2.80 ± 1.98	2.78 ± 1.99	2.83 ± 2.00	0.92
fourth quick flicks	2.89 ± 1.99	2.68 ± 1.99	3.15 ± 1.98	0.28
fifth quick flicks	2.66 ± 1.98	2.62 ± 1.97	2.70 ± 2.00	0.92
first 10-s contractions	1.99 ± 1.69	2.08 ± 1.77	1.88 ± 1.60	0.86
second 10-s contractions	2.27 ± 1.85	2.30 ± 1.88	2.23 ± 1.85	0.89
third 10-s contractions	2.34 ± 1.89	2.22 ± 1.84	2.50 ± 1.96	0.61
fourth 10-s contractions	2.31 ± 1.87	2.30 ± 1.88	2.33 ± 1.89	0.95
fifth 10-s contractions	2.31 ± 1.87	2.28 ± 1.88	2.35 ± 1.87	0.75
60-s static holds	1.74 ± 1.46	1.64 ± 1.34	1.88 ± 1.60	0.71

Values are expressed as M ± SD; BMI, body mass index; IIQ, Incontinence Impact Questionnaire (0–100); *Mann–Whitney test; *p* < 0.05 was considered statistically significant; PFM, pelvic floor muscles; Scale 1–5: (1) PFM activated first in order; (2) PFM activated second; (3) PFM activated third; (4) PFM activated fourth; (5) lack of PFM neuromuscular activity.

The analysis of variance (Friedman ANOVA test) and the Dunn–Bonferroni *post hoc* tests (Table 2) made it possible to compare the differences in changes of the PFM firing order in particular motor tasks presented by both groups. No statistically substantial differences were identified. Surprisingly, the results of all study participants, including the controls, improved the technique of the PFM contractions in the second EMG assessment.

**TABLE 2:** The firing order of the pelvic floor muscles (PFMs) in selected motor tasks before and after a single electromyography (EMG) biofeedback session in the biofeedback and control.

Motor tasks for PFM contractions	Biofeedback group (*n* = 50)		Control group (*n* = 40)		ANOVA *p*-value*
	I EMG assessment	II EMG assessment	I EMG assessment	II EMG assessment	
first quick flicks	2.44 ± 1.94	1.69 ± 1.48	2.75 ± 1.97	2.43 ± 1.92	0.27
second quick flicks	2.6 ± 1.98	2.14 ± 1.73	2.8 ± 2.02	2.5 ± 1.96	0.62
third quick flicks	2.78 ± 1.99	2.08 ± 1.74	2.83 ± 2	2.33 ± 1.89	0.15
fourth quick flicks	2.68 ± 1.99	2.14 ± 1.79	3.15 ± 1.98	2.3 ± 1.9	0.33
fifth quick flicks	2.62 ± 1.97	1.98 ± 1.7	2.7 ± 2	2.4 ± 1.93	0.22
first 10-s contractions	2.08 ± 1.75	1.57 ± 1.35	1.88 ± 1.6	1.83 ± 1.62	0.5
second 10-s contractions	2.29 ± 1.86	2.06 ± 1.65	2.23 ± 1.85	2.43 ± 1.92	0.81
third 10-s contractions	2.2 ± 1.83	1.98 ± 1.64	2.5 ± 1.96	2.43 ± 1.92	0.59
fourth 10-s contractions	2.27 ± 1.87	2.12 ± 1.8	2.33 ± 1.89	2.4 ± 1.93	0.9
fifth 10-s contractions	2.25 ± 1.87	2.08 ± 1.67	2.35 ± 1.87	2.43 ± 1.92	0.82
60-s static holds	1.63 ± 1.33	1.39 ± 1.02	1.88 ± 1.6	1.65 ± 1.37	0.4

Values are expressed as M ± SD; *Friedman ANOVA test; Dunn–Bonferroni *post hoc* tests; *p* < 0.05 was considered statistically significant. PFM, pelvic floor muscles; Scale 1–5: (1) PFM activated first in order; (2) PFM activated second; (3) PFM activated third; (4) PFM activated fourth; (5) lack of PFM neuromuscular activity; EMG, surface electromyography.

In the Table 3, we presented the numbers of participants classified as responders or non-responders, comparing their technique of PFM contractions in the first and second EMG assessments. Based on the mean outcomes from eleven motor tasks, 73% of women in the biofeedback group maintained correct technique or improved it in the second EMG assessment (thus, were classified as responders). In the control group, such performance was observed on average in 65% of participants. What is more, in ten from eleven motor tasks we observed more responders in the biofeedback group than in the control group.

**TABLE 3:** The number of responders and non-responders in teaching pelvic floor muscles (PFM) contraction in a single surface electromyography (EMG) biofeedback session.

Motor tasks for PFM contractions	Group	Responders; *n* (%)	Non-responders; *n* (%)
first quick flicks	Biofeedback	42 (84)	8 (16)
	Control	25 (63)	15 (37)
second quick flicks	Biofeedback	34 (68)	16 (32)
	Control	25 (63)	15 (37)
third quick flicks	Biofeedback	36 (52)	14 (48)
	Control	26 (65)	14 (35)
fourth quick flicks	Biofeedback	35 (70)	15 (30)
	Control	27 (67)	13 (33)
the fifth quick flick	Biofeedback	38 (76)	12 (24)
	Control	26 (65)	14 (35)
first 10-s contractions	Biofeedback	43 (86)	7 (14)
	Control	31 (78)	9 (22)
second 10-s contractions	Biofeedback	34 (68)	16 (32)
	Control	25 (63)	15 (37)
third 10-s contractions	Biofeedback	37 (74)	13 (26)
	Control	25 (63)	15 (37)
fourth 10-s contractions	Biofeedback	36 (72)	14 (28)
	Control	26 (65)	14 (35)
fifth 10-s contractions	Biofeedback	33 (66)	17 (34)
	Control	25 (63)	15 (37)
60-s static holds	Biofeedback	45 (90)	5 (10)
	Control	32 (64)	8 (16)

Responders are participant who maintained correct technique of the PFM contractions or improved it in the second EMG assessment. Non-responders are participant who performed the PFM contractions incorrectly in both EMG assessments or presented worse technique in the second assessment. PFM, pelvic floor muscles; EMG, surface electromyography.

In this study we opted for an alternative approach to qualitative data analysis. All values, from the first quick flicks to static holds, were mapped to the appropriate dichotomous scale. Each study participant received one score, if her PFM were contracted first compared to the synergistic muscles. Those participants, who activated PFM after synergistic muscles or didn’t activated PFM at all, were scored 0. We scored the participant’s performance of the PFM contraction performance separately for the first and second EMG assessments. Based on this aggregated information, we were able to place individuals in the appropriate cell of the contingency table.

Table 4 shows how the scores were allocated using the example of the performance of the first quick flicks by the biofeedback group. At the intersection of the corresponding rows and columns, the contingency table shows the number of participants from the biofeedback group who performed the first quick flicks correctly or incorrectly during the first and second EMG assessments. In this case, there were 30 women who correctly executed the quick flicks in the first and second EMG assessments, 2 women whose results were worse in comparison to the first assessment, 12 improved their scores and 6 didn’t present improvement. We performed the above calculations and classification in the appropriate cell of the contingency table, separately for the biofeedback and control groups. Based on this, we were able to use the Chi-square McNamara B/C test to determine whether a single biofeedback session can statistically improve participants’ performance. We confirmed that statistical significance below the presumed value was recorded for four motor tasks in the biofeedback group: the first quick flicks (*p* = 0.016), the third quick flicks (*p* = 0.027), the fifth quick flicks (*p* = 0.008), and the first 10-s contraction (*p* = 0.046) and at one parameter in the control group: the fourth 10-s contractions (*p* = 0.009). The data analysis of the changes in the PFM contraction technique in other motor tasks in both groups are presented in the Supplementary Tables 5–25.

**TABLE 4:** Sample contingency table, showing the distribution of the first quick flick performance in the first and second electromyograph (EMG) pelvic floor muscles (PFM) assessments in the biofeedback group (*n* = 50).

Performance of the first quick flicks	II EMG assessment (YES)	II EMG assessment (NO)
**I EMG assessment (YES)**	30	2
**I EMG assessment (NO)**	12	6

YES: the number of participants who activated the PFM first in order (correct technique); NO: the number of participants who activated the PFM after synergistic muscles or did not activate PFM at all (incorrect technique); PFMs, pelvic floor muscles; EMG, surface electromyography; analyzed with the Chi-square McNamara B/C test: *p* = 0.016.

## Discussion

The most important finding of our study is that a single EMG biofeedback session is beneficial for the performance of the PFM contractions in pregnant, continent women. Firstly, when applying one-time biofeedback session, more study participants maintained correct technique or improved it (classified as “responders”), compared to the control group. Secondly, in the biofeedback group we observed a statistically significant improvement of PFM firing order in four from 11 motor tasks. These results are in line with our data from a previous work, in which we presented the improvement in the level of neuromuscular activity of PFM after using one-time EMG biofeedback (Bludnicka et al., 2020).

Our outcomes correspond to the conclusion from a systematic review and meta-analysis by Wu et al. (2021) that adding EMG biofeedback to standard PFM training improves its effectiveness in the therapy of pelvic floor dysfunctions. Similar findings were presented also by other authors analyzing relationships between EMG biofeedback and pelvic pain in various populations (Wagner et al., 2022). However, various researchers mainly focused on the effectiveness of multiple biofeedback applications (Batista et al., 2011; de Oliveira Ferro et al., 2020; Kopanska et al., 2020). The novel issue of our study is that we noted substantial positive effects of a single biofeedback session. This observation may have important value for clinical and exercise practice. A single session instead of multiple visits to a urogynecological physiotherapist would certainly reduce the costs of the intervention and make the organization of the treatment easier for the patients.

Very interesting outcomes are those observed in the control group. Although after intervention on average the control women presented worse technique of the PFM contractions than the biofeedback group, a substantial part of them also positively responded to repetition of the EMG assessment. This may justify the assumption of other authors that the vaginal probe might offer strong proprioceptive feedback (Bø and Sherburn, 2007) and that each repetition of the PFM contraction can lead to an improvement in the contraction technique. On the other hand, the better outcomes in the second assessment may be due to practice in activating PFM based on appropriate instructions given by the investigator at the start of the study. Such an interpretation of the data would support the conclusion of other authors that appropriate instructions for women are sufficient to properly activate PFM (Kandadai et al., 2015; Vermandel et al., 2015; Ben Ami and Dar, 2018). Charlanes et al. (2021) concluded that the most comprehensible and acceptable instruction for assessing PFM contractions is the combination of two simple instructions: one anatomical and one functional.

Teaching pregnant women how to perform PFM exercises seems to be particularly important to prevent PFM disorders such UI, both during and after pregnancy (Davenport et al., 2018; Woodley et al., 2020). More and more women would like to continue their exercise programs during pregnancy, even based on the high-impact and high intensity activities (Szumilewicz et al., 2022). Therefore, they should know how to use “the knack”–a quick, strong, well-timed PFM contraction, before and during physical stress increasing intraabdominal pressure (like jumping or running). By activating the PFMs as quickly as possible, it is possible to counteract the increase in pressure in the abdominal cavity, thus effectively reducing urine loss. Using “the knack” together with education on PFM functions and training appeared to be an effective strategy to maintain continence during pregnancy and postpartum, in women attending high-low impact exercise programs (Szumilewicz et al., 2019a,^2020^). Taking it into account, it is very likely that women who are not able to activate PFM in an appropriate moment or who contract synergistic muscles instead of PFM, will not be able to employ “the knack” during their daily or sport activities and will experience urine leakage more often. However, to confirm this thesis, the relationship of firing order and symptoms of UI should be further researched.

Based on the opinions of other authors (Bø and Sherburn, 2007) the simultaneous contractions of synergistic muscles may negatively impact the awareness and strength of the pelvic-floor muscle contraction. According to Neels et al. (2018) contractions of other muscles (rectus abdominis, the gluteal muscles, and the adductors), as well as other movements (pelvic tilt, breath holding, and straining) performed in addition to or instead of the PFM contractions, are probably the most common mistakes when trying to contract the PFM. What is more, an important factor in the prevention of urine leakage is the proper timing of pelvic-floor muscle activity in relation to the activity of other trunk muscles (Moser et al., 2018; Koenig et al., 2021). Therefore, in this study the firing order of PFM contractions in relation to synergistic muscles was the subject of quantitative and qualitative analysis. Using EMG assessment, we were able to assess which muscle group was activated first in order: pelvic floor, abdominal or gluteus muscles. Taking into account the reliability of EMG PFM assessment proved by other authors (Glazer et al., 1999; Scharschmidt et al., 2020), our outcomes are scientifically well founded and appear to be credible for clinical practice.

Surface EMG is normally utilized in the research assessment and treatment when it is intended to quantitatively measure the electrophysiological response of the neuromuscular system. Non-invasive assessment protocols for most muscle groups, despite being internationally standardized, have not yet been approved as a solution for PFMs disorders, making it even more challenging to standardize their scientific research and clinical applicability (de Oliveira Ferro et al., 2020). For the assessment of PFM function, physiotherapists most often use the Perfect and Oxford scales. The name of the scale PERFECT has derived from the first initials of the pelvic floor efficiency tasks assessed (P, power; E, endurance; R, repetitions; F, fast; E, elevation; C, co-contraction; T, timing). Laycock developed the Modified Oxford Grading System to evaluate the strength of the PFMs by vaginal palpation. It consists of a six-point scale: 0 = no contraction, 1 = flicker, 2 = weak, 3 = moderate, 4 = good (with lift), and 5 = strong (Laycock and Jerwood, 2001). In this work, we applied the 1–5 point scale, where “1” meant that the PFM were activated (fired) first in relation to three synergistic muscle groups and “5” that PFM did not activate at all. Both palpation and surface EMG can be a reliable source of data in the research and clinical settings (Botelho et al., 2013; Szumilewicz et al., 2019b). However, EMG assessments offer women more privacy, allowing them to monitor their PFM neuromuscular activity and encouraging them to continue exercising (Chen and Tzeng, 2009).

### Strengths and limitations

The analysis of the firing the order of PFMs after one-time use of biofeedback in pregnant, continent women is a novel idea. The study was conducted in a group of moderate size. Certainly, for a broader generalization of our conclusion, this study requires further implementation, including women of various races, ages, parity, and health conditions. Nevertheless, it seems to provide sufficient evidence for the practical and clinical value of a one-time EMG biofeedback session.

The limitation of our work was that the qualification of study participants was based on the Incontinence Impact Questionnaire scores. Some women, being ashamed, would not report on their PFM dysfunctions, especially on symptoms of UI. In order to be sure of the homogeneity of the research group, it would be worth using objective methods of assessing UI. Another limitation of our work was that we did not analyze the muscle fatigue, which could occur differently in each participant. In particular, this may affect the technique of PFM contraction during the last motor task. The issue of muscle fatigue requires attention in our future research.

## Conclusion

Our study revealed that the one-time EMG biofeedback has a positive effect on the performance of PFM contraction, increasing the chance to activate PFM before synergistic muscles. Given the potential difficulties in the initial performance of PFM contractions and the beneficial impact in this regard of a single EMG biofeedback session, it should be recommended as a standard teaching method for the PFM exercises in pregnant, continent women.

## Data availability statement

The data that support the findings of this study are available from the corresponding author upon reasonable request.

## Ethics statement

The principles of the WMA Declaration of Helsinki were used as guidelines for this study and the approval of the Bioethics Commission at the District Medical Chamber in Gdansk, Poland (KB-8/14) was as well obtained beforehand. The participants were asked to sign informed consent prior to the commencements of tests. The study was retrospectively registered in 2016 with regard to the pilot phase that we carried out in 2013–2015. For the later stages of the project, including the research presented in this study (performed in 2016–2019), this was a prospective registration. The patients/participants provided their written informed consent to participate in this study.

## Author contributions

MB: 45% of study design, data collection, data interpretation, manuscript preparation, and literature search. MP: 10% of study design, data collection, data interpretation, manuscript preparation, and literature search. JK and DB: 5% of statistical analysis. BD-B: 5% of data interpretation and final revision of the text. AS: 30% of study design, data collection, data interpretation, manuscript preparation, literature search, funds collection, and final revision of the text. All authors contributed to the article and approved the submitted version.
