# Supplementary material for: The influence of one-time biofeedback electromyography session on the firing order in the pelvic floor muscle contraction in pregnant woman–A randomized controlled trial
Source: Front Hum Neurosci. 2022 Sep 29;16:944792. doi: 10.3389/fnhum.2022.944792 (PMC9559232; doi:10.3389/fnhum.2022.944792)
Supplement: Supplementary file 1 [file Data_Sheet_1.docx]

# Supplementary Data

| **TABLE 5 \| The contingency table, showing the distribution of the second quick flick performance in the first and second EMG PFM assessments in the biofeedback group** | | | | |
| --- | --- | --- | --- | --- |
| ***Performance of the second quick flicks*** | **II EMG assessment (YES)** | | **II EMG assessment (NO)** |  |
| **I EMG assessment (YES)** | | 26 | 4 |  |
| **I EMG assessment (NO)** | | 8 | 12 |  |
| YES: the number of participants who activated the PFM first in order (correct technique); NO: the number of participants who activated the PFM after synergistic muscles or did not activate PFM at all (incorrect technique); PFM, pelvic floor muscles; EMG, surface electromyography; analyzed with the Chi-square McNemara B/C test: p = 0.386 | | | | |

| **TABLE 6 \| The contingency table, showing the distribution of the third quick flick performance in the first and second EMG PFM assessments in the biofeedback group** | | | | |
| --- | --- | --- | --- | --- |
| ***Performance of the third quick flicks*** | **II EMG assessment (YES)** | | **II EMG assessment (NO)** |  |
| **I EMG assessment (YES)** | | 25 | 2 |  |
| **I EMG assessment (NO)** | | 11 | 12 |  |
| YES: the number of participants who activated the PFM first in order (correct technique); NO: the number of participants who activated the PFM after synergistic muscles or did not activate PFM at all (incorrect technique); PFM, pelvic floor muscles; EMG, surface electromyography; analyzed with the Chi-square McNemara B/C test: p = 0.026 | | | | |

| **TABLE 7 \| The contingency table, showing the distribution of the fourth quick flick performance in the first and second EMG PFM assessments in the biofeedback group** | | | | |
| --- | --- | --- | --- | --- |
| ***Performance of the fourth quick flicks*** | **II EMG assessment (YES)** | | **II EMG assessment (NO)** |  |
| **I EMG assessment (YES)** | | 27 | 2 |  |
| **I EMG assessment (NO)** | | 8 | 13 |  |
| YES: the number of participants who activated the PFM first in order (correct technique); NO: the number of participants who activated the PFM after synergistic muscles or did not activate PFM at all (incorrect technique); PFM, pelvic floor muscles; EMG, surface electromyography; analyzed with the Chi-square McNemara B/C test: p = 0.113 | | | | |

| **TABLE 8 \| The contingency table, showing the distribution of the fifth quick flick performance in the first and second EMG PFM assessments in the biofeedback group** | | | | |
| --- | --- | --- | --- | --- |
| ***Performance of the fifth quick flicks*** | **II EMG assessment (YES)** | | **II EMG assessment (NO)** |  |
| **I EMG assessment (YES)** | | 29 | 0 |  |
| **I EMG assessment (NO)** | | 9 | 12 |  |
| YES: the number of participants who activated the PFM first in order (correct technique); NO: the number of participants who activated the PFM after synergistic muscles or did not activate PFM at all (incorrect technique); PFM, pelvic floor muscles; EMG, surface electromyography; analyzed with the Chi-square McNemara B/C test: p = 0.007 | | | | |

| **TABLE 9 \| The contingency table, showing the distribution of the first contractions performance in the first and second EMG PFM assessments in the biofeedback group** | | | | |
| --- | --- | --- | --- | --- |
| ***Performance of the first contractions*** | **II EMG assessment (YES)** | | **II EMG assessment (NO)** |  |
| **I EMG assessment (YES)** | | 35 | 1 |  |
| **I EMG assessment (NO)** | | 8 | 6 |  |
| YES: the number of participants who activated the PFM first in order (correct technique); NO: the number of participants who activated the PFM after synergistic muscles or did not activate PFM at all (incorrect technique); PFM, pelvic floor muscles; EMG, surface electromyography; analyzed with the Chi-square McNemara B/C test: p = 0.045 | | | | |

| **TABLE 10 \| The contingency table, showing the distribution of the second contractions performance in the first and second EMG PFM assessments in the biofeedback group** | | | | |
| --- | --- | --- | --- | --- |
| ***Performance of the second contractions*** | **II EMG assessment (YES)** | | **II EMG assessment (NO)** |  |
| **I EMG assessment (YES)** | | 26 | 7 |  |
| **I EMG assessment (NO)** | | 8 | 9 |  |
| YES: the number of participants who activated the PFM first in order (correct technique); NO: the number of participants who activated the PFM after synergistic muscles or did not activate PFM at all (incorrect technique); PFM, pelvic floor muscles; EMG, surface electromyography; analyzed with the Chi-square McNemara B/C test: p = 1.000 | | | | |

| **TABLE 11 \| The contingency table, showing the distribution of the third contractions performance in the first and second EMG PFM assessments in the biofeedback group** | | | | |
| --- | --- | --- | --- | --- |
| ***Performance of the third contractions*** | **II EMG assessment (YES)** | | **II EMG assessment (NO)** |  |
| **I EMG assessment (YES)** | | 26 | 7 |  |
| **I EMG assessment (NO)** | | 8 | 9 |  |
| YES: the number of participants who activated the PFM first in order (correct technique); NO: the number of participants who activated the PFM after synergistic muscles or did not activate PFM at all (incorrect technique); PFM, pelvic floor muscles; EMG, surface electromyography; analyzed with the Chi-square McNemara B/C test: p = 0.546 | | | | |

| **TABLE 12 \| The contingency table, showing the distribution of the fourth contractions performance in the first and second EMG PFM assessments in the biofeedback group** | | | | |
| --- | --- | --- | --- | --- |
| ***Performance of the fourth contractions*** | **II EMG assessment (YES)** | | **II EMG assessment (NO)** |  |
| **I EMG assessment (YES)** | | 28 | 5 |  |
| **I EMG assessment (NO)** | | 8 | 9 |  |
| YES: the number of participants who activated the PFM first in order (correct technique); NO: the number of participants who activated the PFM after synergistic muscles or did not activate PFM at all (incorrect technique); PFM, pelvic floor muscles; EMG, surface electromyography; analyzed with the Chi-square McNemara B/C test: p = 0.579 | | | | |

| **TABLE 13 \| The contingency table, showing the distribution of the fifth contractions performance in the first and second EMG PFM assessments in the biofeedback group** | | | | |
| --- | --- | --- | --- | --- |
| ***Performance of the fifth contractions*** | **II EMG assessment (YES)** | | **II EMG assessment (NO)** |  |
| **I EMG assessment (YES)** | | 26 | 8 |  |
| **I EMG assessment (NO)** | | 7 | 9 |  |
| YES: the number of participants who activated the PFM first in order (correct technique); NO: the number of participants who activated the PFM after synergistic muscles or did not activate PFM at all (incorrect technique); PFM, pelvic floor muscles; EMG, surface electromyography; analyzed with the Chi-square McNemara B/C test: p = 1.000 | | | | |

| **TABLE 14 \| The contingency table, showing the distribution of the static holds performance in the first and second EMG PFM assessments in the biofeedback group** | | | | |
| --- | --- | --- | --- | --- |
| ***Performance of the static holds*** | **II EMG assessment (YES)** | | **II EMG assessment (NO)** |  |
| **I EMG assessment (YES)** | | 35 | 3 |  |
| **I EMG assessment (NO)** | | 10 | 2 |  |
| YES: the number of participants who activated the PFM first in order (correct technique); NO: the number of participants who activated the PFM after synergistic muscles or did not activate PFM at all (incorrect technique); PFM, pelvic floor muscles; EMG, surface electromyography; analyzed with the Chi-square McNemara B/C test: p = 0.096 | | | | |

| **TABLE 15 \| The contingency table, showing the distribution of the first quick flick performance in the first and second EMG PFM assessments in the control group** | | | | |
| --- | --- | --- | --- | --- |
| ***Performance of the first quick flicks*** | **II EMG assessment (YES)** | | **II EMG assessment (NO)** |  |
| **I EMG assessment (YES)** | | 18 | 3 |  |
| **I EMG assessment (NO)** | | 7 | 12 |  |
| YES: the number of participants who activated the PFM first in order (correct technique); NO: the number of participants who activated the PFM after synergistic muscles or did not activate PFM at all (incorrect technique); PFM, pelvic floor muscles; EMG, surface electromyography; analyzed with the Chi-square McNemara B/C test: p = 0.342 | | | | |

| **TABLE 16 \| The contingency table, showing the distribution of the second quick flick performance in the first and second EMG PFM assessments in the control group** | | | | |
| --- | --- | --- | --- | --- |
| ***Performance of the second quick flicks*** | **II EMG assessment (YES)** | | **II EMG assessment (NO)** |  |
| **I EMG assessment (YES)** | | 18 | 4 |  |
| **I EMG assessment (NO)** | | 7 | 11 |  |
| YES: the number of participants who activated the PFM first in order (correct technique); NO: the number of participants who activated the PFM after synergistic muscles or did not activate PFM at all (incorrect technique); PFM, pelvic floor muscles; EMG, surface electromyography; analyzed with the Chi-square McNemara B/C test: p = 0.546 | | | | |

| **TABLE 17 \| The contingency table, showing the distribution of the third quick flick performance in the first and second EMG PFM assessments in the control group** | | | | |
| --- | --- | --- | --- | --- |
| ***Performance of the third quick flicks*** | **II EMG assessment (YES)** | | **II EMG assessment (NO)** |  |
| **I EMG assessment (YES)** | | 19 | 2 |  |
| **I EMG assessment (NO)** | | 7 | 12 |  |
| YES: the number of participants who activated the PFM first in order (correct technique); NO: the number of participants who activated the PFM after synergistic muscles or did not activate PFM at all (incorrect technique); PFM, pelvic floor muscles; EMG, surface electromyography; analyzed with the Chi-square McNemara B/C test: p = 0.182 | | | | |

| **TABLE 18 \| The contingency table, showing the distribution of the fourth quick flicks performance in the first and second EMG PFM assessments in the control group** | | | | |
| --- | --- | --- | --- | --- |
| ***Performance of the fourth quick flicks*** | **II EMG assessment (YES)** | | **II EMG assessment (NO)** |  |
| **I EMG assessment (YES)** | | 16 | 1 |  |
| **I EMG assessment (NO)** | | 11 | 12 |  |
| YES: the number of participants who activated the PFM first in order (correct technique); NO: the number of participants who activated the PFM after synergistic muscles or did not activate PFM at all (incorrect technique); PFM, pelvic floor muscles; EMG, surface electromyography; analyzed with the Chi-square McNemara B/C test: p = 0.009 | | | | |

| **TABLE 19 \| The contingency table, showing the distribution of the fifth quick flick performance in the first and second EMG PFM assessments in the control group** | | | | |
| --- | --- | --- | --- | --- |
| ***Performance of the fifth quick flicks*** | **II EMG assessment (YES)** | | **II EMG assessment (NO)** |  |
| **I EMG assessment (YES)** | | 21 | 2 |  |
| **I EMG assessment (NO)** | | 5 | 12 |  |
| YES: the number of participants who activated the PFM first in order (correct technique); NO: the number of participants who activated the PFM after synergistic muscles or did not activate PFM at all (incorrect technique); PFM, pelvic floor muscles; EMG, surface electromyography; analyzed with the Chi-square McNemara B/C test: p = 0. 449 | | | | |

| **TABLE 20 \| The contingency table, showing the distribution of the first contractions performance in the first and second EMG PFM assessments in the control group** | | | | |
| --- | --- | --- | --- | --- |
| ***Performance of the first contractions*** | **II EMG assessment (YES)** | | **II EMG assessment (NO)** |  |
| **I EMG assessment (YES)** | | 26 | 3 |  |
| **I EMG assessment (NO)** | | 5 | 6 |  |
| YES: the number of participants who activated the PFM first in order (correct technique); NO: the number of participants who activated the PFM after synergistic muscles or did not activate PFM at all (incorrect technique); PFM, pelvic floor muscles; EMG, surface electromyography; analyzed with the Chi-square McNemara B/C test: p = 0.723 | | | | |

| **TABLE 21 \| The contingency table, showing the distribution of the second contractions performance in the first and second EMG PFM assessments in the control group** | | | | |
| --- | --- | --- | --- | --- |
| ***Performance of the second contractions*** | **II EMG assessment (YES)** | | **II EMG assessment (NO)** |  |
| **I EMG assessment (YES)** | | 22 | 5 |  |
| **I EMG assessment (NO)** | | 3 | 10 |  |
| YES: the number of participants who activated the PFM first in order (correct technique); NO: the number of participants who activated the PFM after synergistic muscles or did not activate PFM at all (incorrect technique); PFM, pelvic floor muscles; EMG, surface electromyography; analyzed with the Chi-square McNemara B/C test: p = 0.723 | | | | |

| **TABLE 22 \| The contingency table, showing the distribution of the third contractions performance in the first and second EMG PFM assessments in the control group** | | | | |
| --- | --- | --- | --- | --- |
| ***Performance of the third contractions*** | **II EMG assessment (YES)** | | **II EMG assessment (NO)** |  |
| **I EMG assessment (YES)** | | 22 | 3 |  |
| **I EMG assessment (NO)** | | 3 | 12 |  |
| YES: the number of participants who activated the PFM first in order (correct technique); NO: the number of participants who activated the PFM after synergistic muscles or did not activate PFM at all (incorrect technique); PFM, pelvic floor muscles; EMG, surface electromyography; analyzed with the Chi-square McNemara B/C test: p = 0.683 | | | | |

| **TABLE 23 \| The contingency table, showing the distribution of the fourth contractions performance in the first and second EMG PFM assessments in the control group** | | | | |
| --- | --- | --- | --- | --- |
| ***Performance of the fourth contractions*** | **II EMG assessment (YES)** | | **II EMG assessment (NO)** |  |
| **I EMG assessment (YES)** | | 23 | 3 |  |
| **I EMG assessment (NO)** | | 3 | 11 |  |
| YES: the number of participants who activated the PFM first in order (correct technique); NO: the number of participants who activated the PFM after synergistic muscles or did not activate PFM at all (incorrect technique); PFM, pelvic floor muscles; EMG, surface electromyography; analyzed with the Chi-square McNemara B/C test: p = 0.683 | | | | |

| **TABLE 24 \| The contingency table, showing the distribution of the fifth contractions performance in the first and second EMG PFM assessments in the control group** | | | | |
| --- | --- | --- | --- | --- |
| ***Performance of the fifth contractions*** | **II EMG assessment (YES)** | | **II EMG assessment (NO)** |  |
| **I EMG assessment (YES)** | | 22 | 3 |  |
| **I EMG assessment (NO)** | | 3 | 12 |  |
| YES: the number of participants who activated the PFM first in order (correct technique); NO: the number of participants who activated the PFM after synergistic muscles or did not activate PFM at all (incorrect technique); PFM, pelvic floor muscles; EMG, surface electromyography; analyzed with the Chi-square McNemara B/C test: p = 0.683 | | | | |

| **TABLE 25 \| The contingency table, showing the distribution of the static holds performance in the first and second EMG PFM assessments in the control group** | | | | |
| --- | --- | --- | --- | --- |
| ***Performance of the static holds*** | **II EMG assessment (YES)** | | **II EMG assessment (NO)** |  |
| **I EMG assessment (YES)** | | 26 | 3 |  |
| **I EMG assessment (NO)** | | 6 | 5 |  |
| YES: the number of participants who activated the PFM first in order (correct technique); NO: the number of participants who activated the PFM after synergistic muscles or did not activate PFM at all (incorrect technique); PFM, pelvic floor muscles; EMG, surface electromyography; analyzed with the Chi-square McNemara B/C test: p = 0.505 | | | | |
